# Reverse Engineering Applied to Red Human Hair Pheomelanin Reveals Redox-Buffering as a Pro-Oxidant Mechanism

**DOI:** 10.1038/srep18447

**Published:** 2015-12-16

**Authors:** Eunkyoung Kim, Lucia Panzella, Raffaella Micillo, William E. Bentley, Alessandra Napolitano, Gregory F. Payne

**Affiliations:** 1 Institute for Biosystems and Biotechnology Research University of Maryland 5115 Plant Sciences Building College Park, MD 20742, USA; 2 Fischell Department of Bioengineering University of Maryland College Park, MD 20742, USA; 3 Department of Chemical Sciences, University of Naples Federico II, Via Cintia 4, I-80126 Naples (Italy); 4 Department of Clinical Medicine and Surgery, University of Naples “Federico II” – Via Pansini 5, I-80131 Naples, Italy

## Abstract

Pheomelanin has been implicated in the increased susceptibility to UV-induced melanoma for people with light skin and red hair. Recent studies identified a UV-independent pathway to melanoma carcinogenesis and implicated pheomelanin’s pro-oxidant properties that act through the generation of reactive oxygen species and/or the depletion of cellular antioxidants. Here, we applied an electrochemically-based reverse engineering methodology to compare the redox properties of human hair pheomelanin with model synthetic pigments and natural eumelanin. This methodology exposes the insoluble melanin samples to complex potential (voltage) inputs and measures output response characteristics to assess redox activities. The results demonstrate that both eumelanin and pheomelanin are redox-active, they can rapidly (sec-min) and repeatedly redox-cycle between oxidized and reduced states, and pheomelanin possesses a more oxidative redox potential. This study suggests that pheomelanin’s redox-based pro-oxidant activity may contribute to sustaining a chronic oxidative stress condition through a redox-buffering mechanism.

Melanin is a ubiquitous pigment generally classified as either the black-brown eumelanin or the reddish pheomelanin. Both melanin types are generated by tyrosinase-catalyzed oxidation of tyrosine with the difference being that cysteine intervenes in pheomelanin biosynthesis and is incorporated in the final pigment as illustrated by the putative structures in Fig. 1a^1^,^2^. Traditionally, eumelanin has been considered to perform a UV-protection function^3^ whereas pheomelanin has been implicated in the enhanced melanoma susceptibility of people with red hair and fair skin^4^ due to the capacity of this pigment to act as a potent photosensitizer leading to intense production of reactive oxygen species (ROS) in response to UV-visible light irradiation^5^,^6^. Recent studies with animal models provide evidence that pheomelanin pigment pathway is implicated in *UV radiation-independent* carcinogenic contributions to melanomagenesis by mechanisms of oxidative damage^6^,^7^,^8^ Two non-exclusive hypotheses were proposed to define pheomelanin’s pro-oxidant activity: (i) the pigment promotes the formation of damaging ROS and (ii) the pigment depletes protective antioxidants (e.g., glutathione)^9^,^10^. Chemical studies support these hypotheses and indicate that natural pheomelanins and model synthetic pigments can sustain autooxidation of cellular antioxidants (e.g., glutathione and NADH) in the presence of oxygen with the concomitant production of ROS^9^,^10^.

The challenge in understanding melanin function is that these pigments are structurally complex and there are comparatively few characterization methods available. Recent advances are resolving the functional importance of melanin’s hierarchical structure at the molecular level (i.e., carboxyl functionality)^11^, macromolecular level (aggregate vs polymer)^12^,^13^, and nanoscale (core surface coating)^14^,^15^,^16^,^17^. Importantly, these recent advances have been enabled by the development and application of new investigational methods (e.g., electron paramagnetic resonance spectroscopy, photoelectron emission microscopy, ultrafast spectroscopy)^15^,^18^,^19^,^20^,^21^.

Here, we apply an electrochemical reverse engineering methodology to probe the redox-properties of pheomelanin in comparison with model synthetic pigments and eumelanin. As illustrated in Fig. 1b, there are four features of this electrochemical methodology. First, an insoluble melanin sample is localized near an electrode surface by entrapping the sample in a thin, non-conducting hydrogel film adjacent to the electrode. Second, the insoluble sample is probed using soluble mediators that can shuttle electrons between the sample and the underlying electrode. The use of mediators provides redox connectivity between the melanin sample and electrode and this overcomes the major limitation of electrochemical analysis of insoluble samples^22^. Third, the melanin sample is exposed to a defined sequence of electrical potentials by applying controlled input voltages to the underlying electrode (the soluble mediators serve to transmit the applied potentials to the sample). Fourth, the measured output currents are analyzed to assess the redox activities of the entrapped melanin^23^,^24^. In essence, this reverse engineering approach aims to characterize the redox-properties of the melanin by exposing the sample to controlled redox perturbations and analyzing the output response characteristics. Such reverse engineering approaches are well developed for characterizing the functionality of complex technological systems (e.g., microelectronic devices) and there has been recent interest in developing reverse engineering as a systems biology tool^25^,^26^,^27^,^28^,^29^,^30^,^31^.

In this study, we first apply this experimental reverse engineering approach to compare the redox properties of pheomelanin and eumelanin using model synthetic pigments prepared by oxidation of the biosynthetic precursors under physiologically relevant conditions. We observe that both model melanins are redox-active and can engage in oxidative and reductive redox-cycling reactions. Further, the synthetic pheomelanin was observed to have a more oxidative redox potential than the synthetic eumelanin. We next performed confirmatory studies with natural eumelanin and pheomelanin samples. These natural melanins display characteristics that are intermediate between the synthetic models^32^,^33^,^34^. Independent measurements confirm that the natural melanins are redox-active and that pheomelanin displays a greater redox-based pro-oxidant activity. We suggest a redox buffering mechanism may explain pheomelanin’s pro-oxidative activities.

## Results

### Initial Characterization

Synthetic models of the pheomelanin and eumelanin pigments were investigated in the present study along with natural melanin samples^35^. The synthetic melanins were prepared by enzymatic oxidation of dihydroxyphenylalanine (dopa) and 5-S-cysteinyldopa the main biosynthetic precursors of natural eumelanin and pheomelanin, respectively. Natural melanin samples were purified from human hair by enzymatic proteolytic procedures^9^.

#### Chemical Characterization

Chemical characterization of the melanin-chitosan films were performed using Raman spectroscopy (Figure S1 of Supplementary Information shows SEM images for these films). Figure 2a shows that the spectrum of synthetic eumelanin powder has two closely-spaced characteristic bands^36^. Band 1 (1360 cm^−1^) and band 2 (1580 cm^−1^) that have been attributed to the linear stretching of the C-C bonds within the aromatic rings and the in-plane stretching of the aromatic rings, respectively^36^,^37^. These two characteristic bands were also observed when synthetic eumelanin was entrapped in the chitosan film (chitosan shows no bands in this spectral region).

Raman characterization of the synthetic pheomelanin powder and the film-entrapped pheomelanin are shown in Fig. 2b. Compared to the spectrum for synthetic eumelanin, the Raman spectrum for these pheomelanin samples show two additional prominent peaks above 2000 cm^−1^ (band 3 and band 4). Previous studies suggest these bands are related to overtone or combination bands^38^. These Raman bands were also observed for both the powder and film-entrapped synthetic pheomelanin samples.

Raman spectra of natural melanin samples (Figure S2 of Supplementary Information) exhibit features that are consistent with those observed for synthetic pigments though more complex due to the contribution of the protein matrix^36^,^38^. In the case of natural pheomelanin bands attributable to eumelanin can be identified^36^, suggesting the presence of a significant eumelanin component in natural pheomelanin^39^.

### Reverse Engineering of Synthetic Melanins

#### Initial Characterization to Identify Redox Activity

An initial electrochemical characterization of the synthetic melanins was performed by immersing the film-coated electrodes in a mixed solution of three redox mediators (100 μM Ir^3+^, 50 μM Fc, and 50 μM Ru^3+^) and applying a cyclic potential to the underlying electrode (between −0.5 V and +0.8 V at a scan rate 2 mV/s). The mediator concentrations used in these studies were selected based on initial measurements that served to “tune” conditions to obtain useful output signals. The results from the chitosan coated electrodes with entrapped eumelanin (91 μg/mL) or pheomelanin (104 μg/mL) were compared to a control chitosan-coated electrode (without entrapped melanin). The cyclic voltammogram (CVs) in Fig. 3a,b shows four major regions of interest.

**Figure 1:**
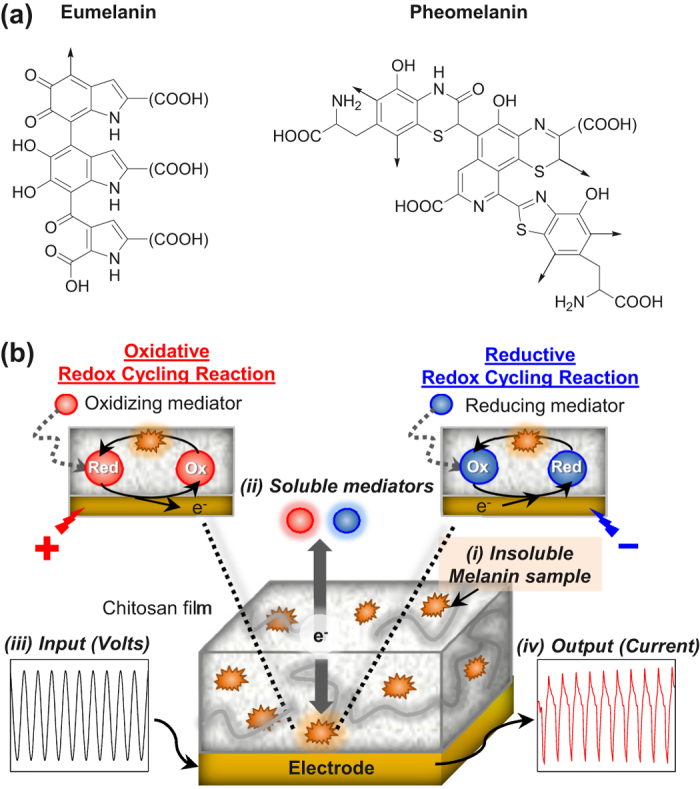
(**a**) Putative structures of eumelanin and pheomelanin. (**b**) Electrochemical approach to reverse engineer melanin: (i) insoluble melanin sample is entrapped in non-conducting film adjacent to electrode; (ii) soluble mediators shuttle electrons between electrode and sample; (iii) complex input potentials (voltages) are applied; and (iv) measured output currents are analyzed.

**Figure 2:**
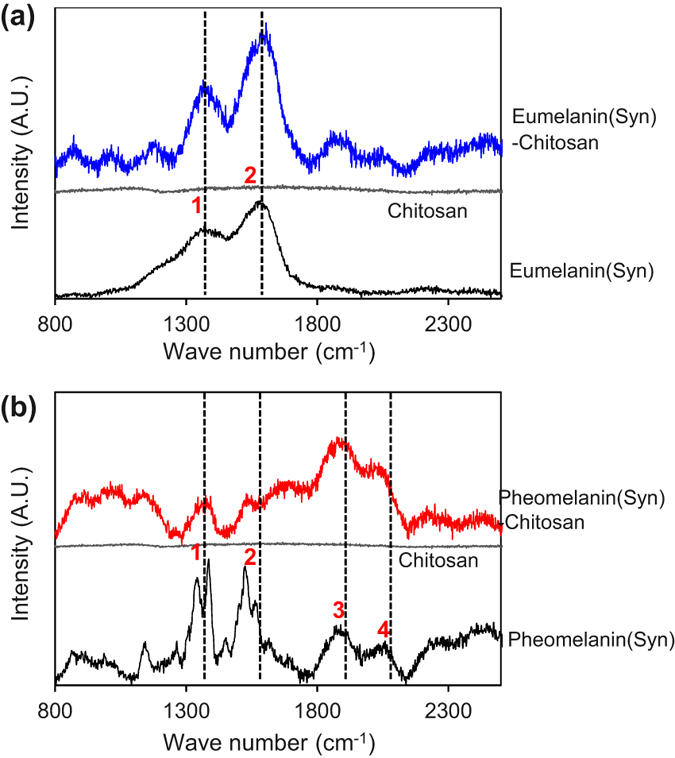
Raman characterization of synthetic melanin samples and melanin-chitosan films. (**a**) Synthetic eumelanin. (**b**) Synthetic pheomelanin.

**Figure 3:**
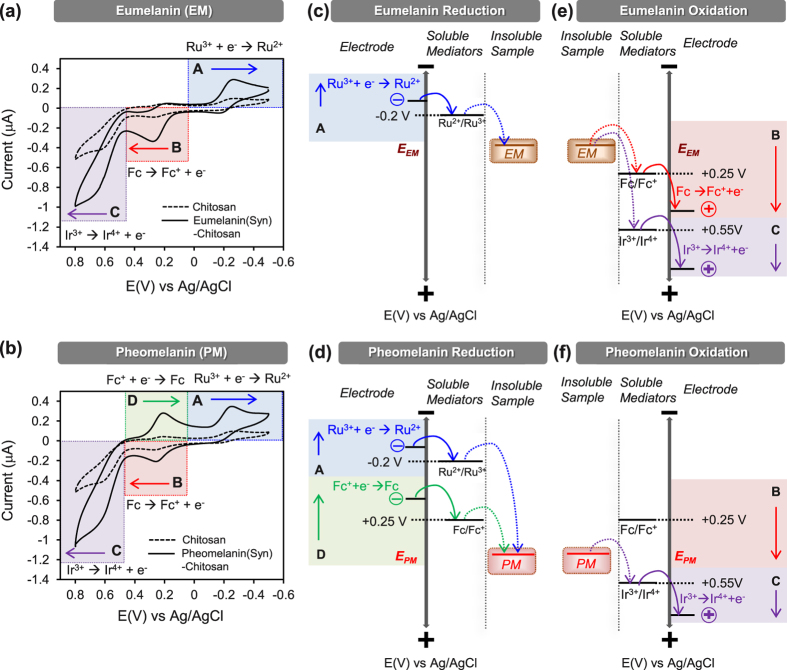
Initial electrochemical characterization of synthetic melanins. Cyclic voltammograms of films containing (**a**) synthetic eumelanin samples and (**b**) synthetic pheomelanin samples compared with the control chitosan films (scan rate of 2 mV/s) in the presence of 3 mediators. (**c**,**d**) Thermodynamic plots illustrating electron exchange between the synthetic melanins and the 3 mediators for reductive redox-cycling reaction. The D region is unique to pheomelanin. (**e**,**f**) Thermodynamic plots for oxidative-redox cycling reaction between synthetic melanins and 3 mediators.

In the “A” region of Fig. 3a,b, the potential was swept in a reducing direction from 0 V to −0.5 V vs Ag/AgCl and the electrode acted as a source of electrons. In this potential range, the mediator Ru^3+^ is electrochemically reduced to Ru^2+^ generating a positive (reducing) current. For the control chitosan-coated electrode, a small reducing current is observed and this current is attributed to the electrochemical reduction of Ru^3+^ ions that diffuse through the film to the electrode. Fig. 3a,b shows considerable amplification of the reducing currents in region A for both melanin models. This amplification is explained by the reductive redox-cycling mechanism in the upper right of Fig. 1b. Specifically, the electrochemically-reduced Ru^2+^ species diffuses into the film and transfers its electrons to the entrapped melanin thereby converting the oxidized melanin moieties into their reduced states and regenerating the oxidized Ru^3+^ mediator. Thus, the observed output response in region A (amplified Ru^3+^ reduction current) indicates that the incorporation of either synthetic melanin confers redox activity to the films.

In the “B” region, the potential is swept through a slightly positive potential allowing the electrode to serve as a sink for electrons. Under this condition, the oxidizing mediator Fc donates electrons to the electrode to generate the negative (oxidative) current. Compared to the chitosan control, the eumelanin-chitosan coated electrode (Fig. 3a) shows a considerable amplification of the oxidizing current in region B which is explained by the oxidative redox-cycling mechanism in the upper left of Fig. 1b. In this case, the electrochemically-oxidized Fc^+^ species is reduced in the film by accepting electrons from the entrapped eumelanin (thereby converting reduced eumelanin moieties into their oxidized states). The electrode coated with the pheomelanin-chitosan film (Fig. 3b) shows less amplification in this B region compared to the eumelanin sample.

In the “C” region, the potential is swept to a more highly positive potential to allow oxidation of another oxidizing mediator Ir^3+^. Under these more oxidative conditions, Ir^3+^ can be oxidized at the electrode to generate a negative current. Figure 3a,b shows oxidation currents in this C region are amplified for both the eumelanin and pheomelanin films. Analogous to the arguments above for the B region, the amplified oxidative currents in region C result from an oxidative redox-cycling mechanism in which Ir^3+^ shuttles electrons from the entrapped melanin to the electrode. Again, the observed output response (amplified oxidation currents in regions B and C) indicate that melanin confers redox-activity to the films.

In the “D” region, the potential is swept in the reducing direction from +0.4 V to 0 V which allows oxidized Fc^+^ to be electrochemically reduced to Fc. Figure 3b shows that amplified output currents are observed in region D for the pheomelanin but not the eumelanin samples (Fig. 3a).

The first important response characteristic observed in Fig. 3 is the paired amplification of oxidation and reduction currents which provides evidence that both synthetic melanin models are redox-active. Specifically, paired amplifications of oxidation and reduction currents is a characteristic signature of redox-cycling^24^ and suggests that the melanins can participate in both oxidative and reductive redox-cycling by being switched to a reduced state (by accepting electrons from the mediators; regions A or D) and to an oxidized state (by donating electrons to mediators; regions B and C).

A second important response characteristic of Fig. 3 is that the paired amplifications occur at different potentials which suggest that the synthetic eumelanin and pheomelanin are oxidized/reduced at different potentials. Thermodynamics constrains redox reactions such that electrons “flow” from species with more negative redox potentials to species with more positive redox potentials. This thermodynamic constraint along with the experimental observations is used to estimate reducing potentials for eumelanin (Fig. 3c) and pheomelanin (Fig. 3d) and similarly to estimate oxidation potentials (Fig. 3e,f). Eumelanin has one region of amplified reduction (region A) and two regions of amplified oxidation (regions B and C), indicating that the reduction-oxidation (redox) potential of the entrapped eumelanin is bracketed at the reducing end by the *E°* of Ru^3+^ (−0.2 V vs Ag/AgCl) and at the oxidizing end by the *E°* of Fc (+0.25 V). The distinctive output response of the film-entrapped pheomelanin sample (amplification of region D) indicates it can undergo a reductive redox-cycling by accepting electrons from Fc (and then donating them to Ir^3+^). This provides initial evidence that pheomelanin is a stronger oxidant with a redox potential that is bracketed at the reducing end by the *E°* of Fc (+0.25 V) and at the oxidizing end by the *E°* of Ir^3+^ (+0.55 V).

Table 1 suggests putative redox reactions for these melanin samples. We propose eumelanin’s redox-activity involves the oxidation of 5,6-dihdroxyindole groups to form extended quinones^40^. We propose pheomelanin’s redox-activity is related to the benzothiazine groups of pheomelanin and putative reactions are suggested in Table 1^41^,^42^,^43^. [Note: as discussed later pheomelanin may have an additional redox activity, between −0.2 V and +0.25 V.]

It is important to note that the observed electrochemical response characteristics indicate that both synthetic melanins are redox-active. Electrochemical measurements over prolonged times (Figure S3 of Supplementary Information) indicate that these redox activities are stable and the melanin can be repeatedly oxidized and reduced. Thus, while electrochemistry provides no chemical structural information of the individual moieties (quinone, semi-quinone, and hydroquinone)^32^,^44^, it does indicate that the distribution among these states is dynamic depending on the environmental context.

#### Estimating Redox Potential

To confirm initial estimates of the redox potentials for the synthetic melanins, we probed the samples with two redox mediators to detect output responses (paired amplification of oxidation and reduction currents) that characterize redox-cycling. Sustained redox-cycling requires at least two mediators and the mediators’ redox potentials must bracket the sample’s redox potential. Thus, this test allows estimation of a sample’s redox potential.

**Figure 4:**
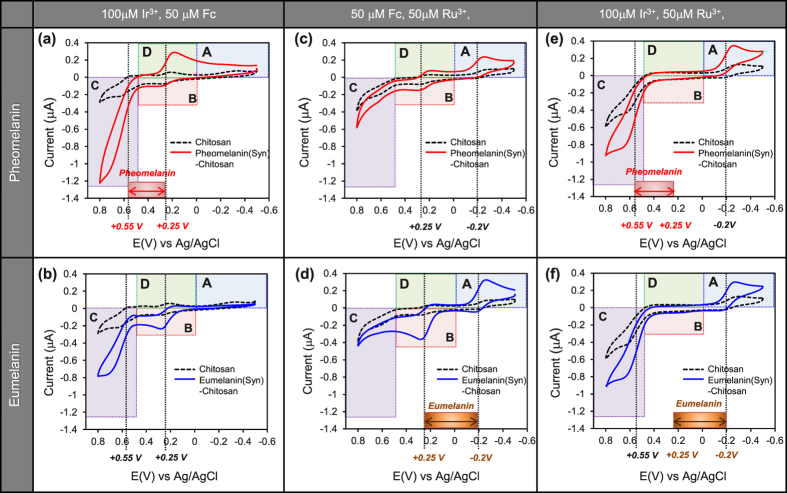
Probing redox potential regions for signatures of redox-cycling (2 mediators). Cyclic voltammograms (CVs) for (**a**,**c**,**e**) synthetic pheomelanin-chitosan films, and (**b**,**d**,**f**) synthetic eumelanin-chitosan films (scan rate of 2 mV/s). Pheomelanin shows redox activity at a more oxidative potential compared to eumelanin.

Figure 4a shows the current-potential curves for the pheomelanin-chitosan and the control chitosan coated electrode in a mixed solution of 100 μM Ir^3+^ and 50 μM Fc. By comparison with the chitosan control, the pheomelanin sample shows a paired amplification of reduction currents in region D and oxidation currents in region C. This paired amplification suggests that pheomelanin can undergo redox-cycling with Fc serving as the reducing agent and Ir^3+^ serving as the oxidant. For the case of eumelanin, Fig. 4b shows no paired amplification as no amplified reduction is apparent. These results confirm that the pheomelanin but not eumelanin model has redox-activity in the potential range bracketed by the *E°*s of Ir^3+^ and Fc (+0.25 V ∼ +0.55 V).

We next compared the control chitosan and pheomelanin-chitosan films using the Fc-Ru^3+^ mediator pair. Figure 4c shows small amplifications in the reducing currents in region A (Ru^3+^) and oxidation currents in region B (Fc). The paired amplification of oxidation and reduction currents provides evidence that the synthetic pheomelanin may have additional redox-activity between −0.2 V and +0.25 V. For the case of eumelanin, Fig. 4d shows substantial paired amplifications (Ru^3+^ reduction in region A and Fc oxidation in region B) confirming that eumelanin is redox-active with a redox potential between −0.2 V and +0.25 V.

Finally, we compared the control chitosan and the melanin-chitosan films using the Ir^3+^ -Ru^3+^ mediator pair that spans the largest range of redox potentials (between −0.2 V and +0.55 V). As indicated at the bottom in Fig. 4e,f, the broad redox range being probed by the Ir^3+^ -Ru^3+^ mediator pair spans the narrower redox windows in which redox activity was observed for pheomelanin (Fig. 4a) and eumelanin (Fig. 4d). Because Fc was deleted from this mediator mixture, Fig. 4e,f show no peaks are observed in region B (Fc oxidation) or region D (Fc reduction). Nevertheless, both the pheomelanin and eumelanin samples showed strong paired amplifications over large potential region which further supports the conclusion that both synthetic melanins have redox activity.

#### Reversibility of Pheomelanin-Specific Oxidative Redox-Activity

**Figure 5:**
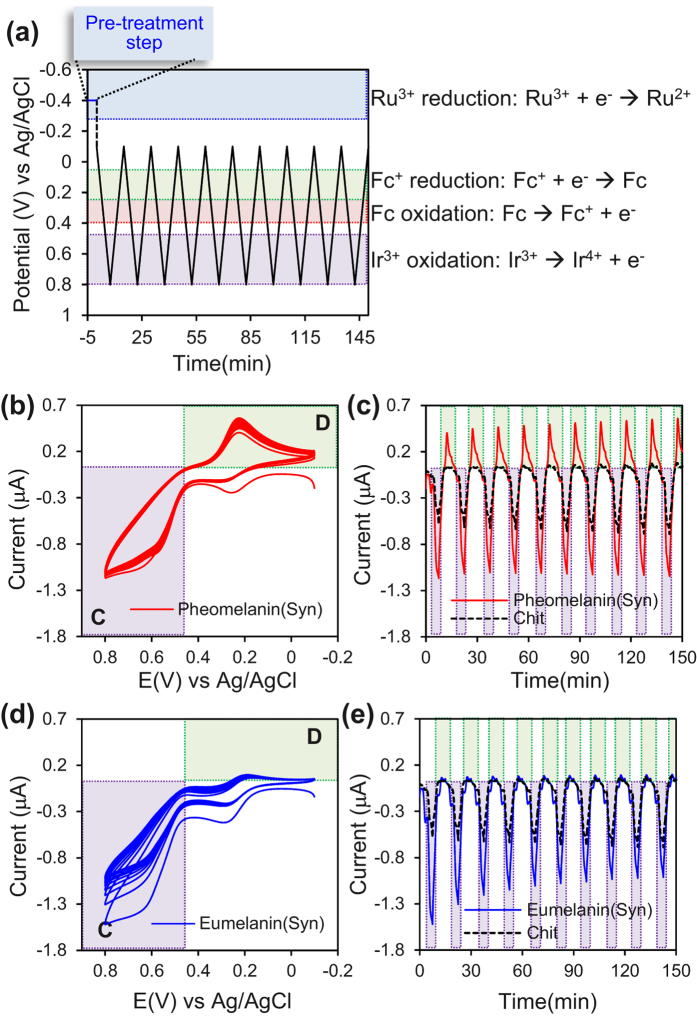
Repeated probing of the oxidative potential window for evidence of synthetic pheomelanin’s reversible redox-activity. (**a**) Sequence of input voltages used to probe the pheomelanin’s oxidative potential window. Current output for pheomelanin-chitosan film expressed as (**b**) cyclic voltammogram or (**c**) output curve. Current output for eumelanin-chitosan film expressed as (**d**) cyclic voltammogram or (**e**) output curve. Pheomelanin’s paired amplification of oxidation-reduction currents and the “steady” output indicates pheomelanin’s oxidative redox activity is reversible.

We next focused on the oxidative potential region to provide further evidence for pheomelanin’s redox activity. Experimentally, we immersed melanin-chitosan coated electrodes in solutions containing all three mediators, and imposed the potential input illustrated in Fig. 5a. The initial “pre-treatment” (−0.4 V vs Ag/AgCl for 5 min) engages the Ru^3+^ mediator in its reductive redox-cycling to transfer electrons from the electrode to the melanin sample (i.e., to reduce some of the redox-active moieties). We then probed the sample by repeatedly cycling the potential between −0.1 V and +0.8 V which precludes the Ru^3+^ mediator from further participating in the redox reactions (i.e., the Ru^3+^ mediator remains “silent” during this probing step).

Figure 5b shows the current response of pheomelanin-chitosan coated electrode to the cyclic imposed potential displayed as a standard cyclic voltammagram (CV), while Fig. 5c shows the results as an output curve (for clarity, the results for the control chitosan film are only shown in Fig. 5c). There are two important observations from these plots. First, the amplification of Ir^3+^ -oxidation (region C) is paired with an amplification of Fc-reduction (region D). Second, both amplifications appear nearly “steady” over time: specifically, the CV curves of Fig. 5b are approximately superimposable and the output curves of Fig. 5c appear to be similar for each consecutive cycle (Note: Figure S4 of the Supplementary Information shows that the output is not exactly steady but rather there is a small net oxidative charge transfer during each cycle). This nearly steady amplification demonstrates the pheomelanin’s oxidative redox activity involves moieties that can be repeatedly oxidized and reduced.

The results with synthetic pheomelanin can be contrasted with those for synthetic eumelanin observed in Fig. 5d,e. Specifically, the amplified oxidation current is not paired with an amplified reduction current indicating a net depletion of electrons from the melanin sample. Consistent with this explanation is the observation that the output for eumelanin is not steady: the CV curves do not superimpose (Fig. 5d) and the peak oxidation currents are attenuated with each consecutive cycle (Fig. 5e). These observations indicate that the electrons initially transferred to the eumelanin sample by Ru^3+^ pre-treatment are being depleted during each Ir^3+^ -oxidation step.

#### Dynamic Analysis: Varying the Input Frequency

**Figure 6:**
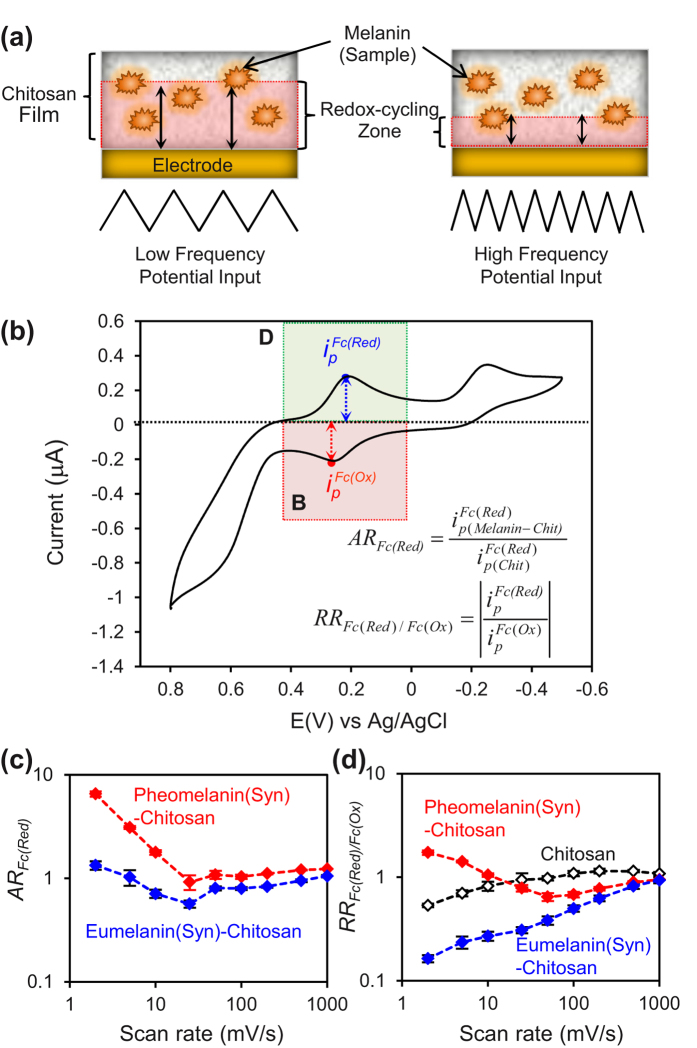
Dynamic probing of the synthetic pheomelanin’s oxidative potential window at varying scan rates. (**a**) Schematic illustrating that scan rate affects the depth of film being probed. (**b**) Parameter ratios used for characterizing redox cycling in the pheomelanin’s oxidative potential window (illustrative CV scan for pheomleanin-chitosan film; 2 mV/s). (**c**) Amplification ratio shows the greatest difference from control chitosan film is observed at the low scan rates (as expected). (**d**) Rectification ratios shows that Fc serves as an oxidant with eumelanin (RR_Fc_ < 1) but a reductant for pheomelanin (RR_Fc_ > 1).

Dynamic analysis is routinely used to reverse engineer technological systems. Figure 6a illustrates that we dynamically probed the film-entrapped melanin samples by varying the frequency of the imposed oscillating potential input (potential range from −0.5 V to +0.8 V; scan rate range 2 to 1000 mV/s) in the presence of all three mediators (Ir^3+^, Fc, and Ru^3+^). As illustrated in Fig. 6a, the input frequency controls the depth of the film region probed by the mediators’ redox-cycling. For analysis, Fig. 6b shows that we focused on the signals associated with Fc reduction (region D) and Fc oxidation (region B) and used two ratios of peak currents to correlate the results. The first ratio, amplification ratio (AR), compares the response of the melanin-containing films to the response of the chitosan film. As expected, Fig. 6c shows that this amplification ratio is greater at lower scan rates while this ratio approaches 1 at high scan rates (i.e., the sample-containing films and control chitosan film behave similarly).

The second ratio is an operational measure of rectification. Figure 6d shows that at high frequencies, the RR_Fc_ for the two melanin samples and the chitosan control all approach 1. At low frequencies, Fig. 6d shows RR > 1 for pheomelanin but RR < 1 for eumelanin which is consistent with previous observations. Specifically, pheomelanin engages Fc in redox-cycling with Fc serving as the reductant (region D) while eumelanin engages Fc in redox-cycling with Fc serving as an oxidant (region B).

#### Dynamic Analysis: Imposing Step Inputs

**Figure 7:**
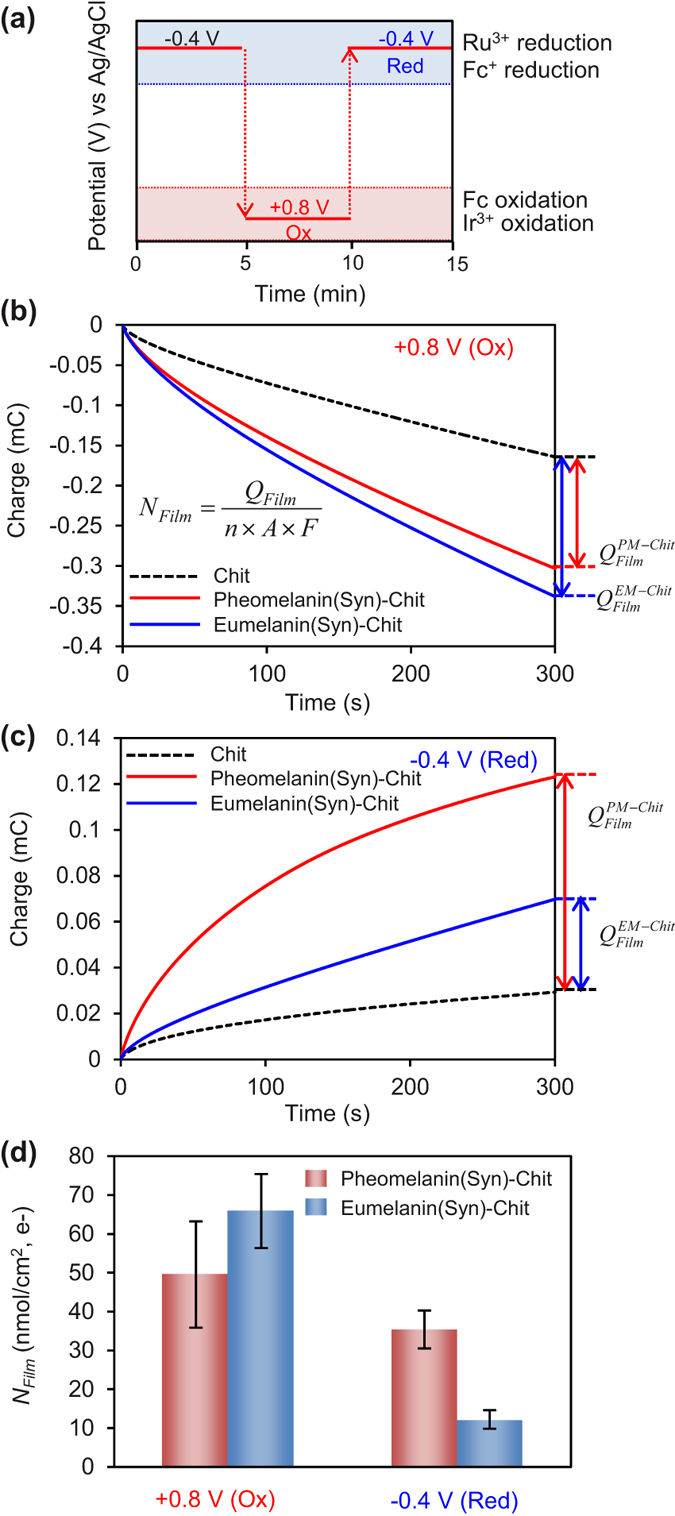
Imposing step potential changes to probe charge transfer. (**a**) Sequence of step changes allows probing for oxidation and reduction. (**b**) Charge transfer observed after a step change for oxidation (*Q*_*Film*_ and *N*_*Film*_ are quantitative measures of electron transfer). (**c**) Charge transfer observed after step change for reduction. (**d**) Summary of *N*_*Film*_ values for various oxidation-reduction steps with synthetic melanins.

A final electrochemical test was performed using a sequence of step potential changes in the presence of all three mediators. Figure 7a shows an initial voltage of −0.4 V was used to initiate Ru^3+^ redox-cycling to transfer electrons to reduce the film-entrapped melanin sample. The first step change from −0.4 to +0.8 V allows both Fc and Ir^3+^ to be oxidized and initiate oxidative redox-cycling to shuttle electrons from the entrapped melanin to the electrode. As indicated in Fig. 7a, this potential was maintained for 5 minutes over which time the electron transfer was measured as shown in Fig. 7b. The cumulative charge transfer (after 5 min) for the control chitosan film was attributed to simple diffusion of the mediators through the film and this value was subtracted from the cumulative charge transfer measured for the films containing the melanin sample. This difference (*Q*_*Film*_) was attributed to redox-cycling in the film and serves as a measure of the number of electrons transferred from the melanin. In principle, this methodology could be used to determine the total redox capacity of the melanin sample, however a 5 minute period is insufficient time to exhaustively transfer electrons from particulate samples (e.g., multiple hours would be required)^45^. Thus, our calculated redox capacity (*N*_*Film*_; nmole electron/cm^2^) is an operational measure useful for semi-quantitative comparison.

Figure 7a shows that after the initial oxidation step, a reduction step was imposed from +0.8 to −0.4 V. This step allows a Ru^3+^ redox-cycling to reduce the melanin by shuttling electrons from the electrode to the melanin. This potential step also allows Fc^+^ to reductively-redox cycle with pheomelanin. Figure 7c shows results from this reduction step and indicates that the charge transfer to both melanin samples is greater than the charge transfer observed with the control film. The ability of these samples to both donate electrons (Fig. 7b) and accept electrons (Fig. 7c) supports the conclusion that both synthetic melanin samples are redox active.

Figure 7d summarizes results from these experiments. The entries in Fig. 7d are for the oxidation experiments of Fig. 7b and reduction experiments of Fig. 7c, respectively. The quantitative differences in *N*_*Film*_ values between these conditions presumably reflect kinetic differences. For instance, the driving force for eumelanin oxidation (by both Ir^3+^ and Fc) is considerably larger than the driving force for eumelanin reduction (by Ru^3+^) and this may explain eumelanin’s larger *N*_*Film*_ in the oxidation step (65 nmole/cm^2^) compared to its reduction step (12 nmole/cm^2^). Similar arguments may explain the differences observed between eumelanin and pheomelanin reduction in the second entry in Fig. 7d: the driving force for pheomelanin’s reduction (by both Ru^3+^ and Fc^+^) is greater than for eumelanin’s reduction (by Ru^3+^).

### Evaluation of Natural Melanin Samples

**Figure 8:**
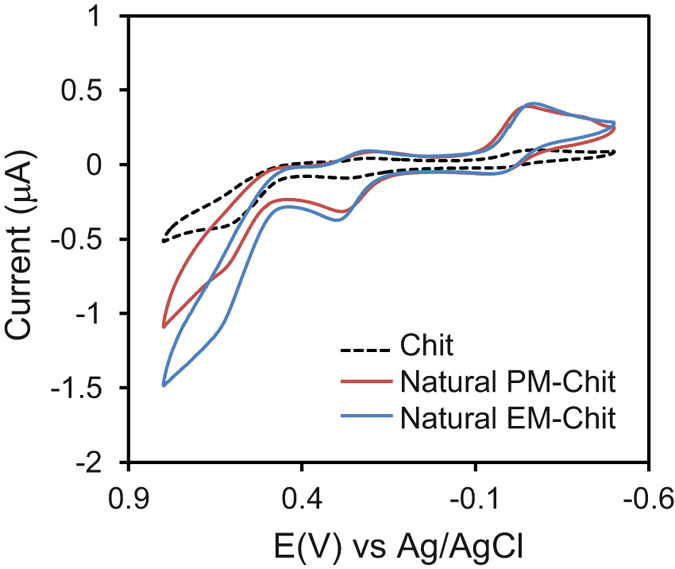
Evidence that natural melanins are redox-active. Cyclic voltammograms of films containing melanin samples (scan rate of 2 mV/s) in the presence of 3 mediators. The paired amplification in oxidation and reduction currents provides evidence that the natural melanins are redox-active and can undergo both oxidative and reductive redox-cycling.

We next evaluated natural eumelanin and pheomelanin samples to provide evidence for the two major observations from studies with the synthetic models: (i) melanin is redox-active; and (ii) pheomelanin has a greater redox-based pro-oxidant activity. In these studies, we prepared melanin-chitosan films with melanin contents of 248 μg/mL and 484 μg/mL for natural eumelanin and pheomelanin, respectively. We immersed the film-coated electrode into a solution containing the three redox mediators (100 μM Ir^3+^, 50 μM Fc, and 50 μM Ru^3+^) and applied varying potential inputs. In the first study, a cyclic potential input (−0.5 to +0.8 V vs Ag/AgCl; scan rate of 2 mV/s) was imposed and Fig. 8 shows significant amplifications in oxidation and reduction currents for both the eumelanin and pheomelanin samples. The paired amplification of oxidation and reduction currents supports the first conclusion that both natural melanins are redox-active and can be switched between reduced and oxidized states.

**Figure 9:**
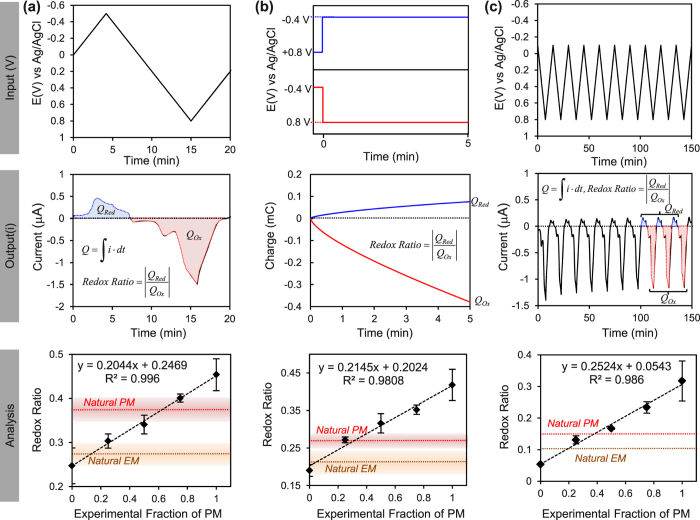
Evidence that natural pheomelanin has a greater redox-based pro-oxidant activity. Three different input potentials were imposed on samples (upper panels), data were analyzed as a ratio of reductive to oxidative charge transfer (middle panels), and results with natural melanin were compared to results for mixtures of synthetic eumelanin and synthetic pheomelanin. (**a**) Cyclic voltammetry. (**b**) Chronocoulometry. (**c**) Cyclic voltammetry for multiple cycles.

Interestingly, the characteristic signature of the synthetic pheomelanin - amplification in the D region observed in Fig. 3 - is not apparent with the natural pheomelanin sample. Possibly, an attenuation of this characteristic D peak may reflect the mixed polymeric nature of the natural melanins. One experimental approach to accommodate this mixed polymeric nature is to compare the behavior of natural melanin samples against a mixture of synthetic melanins^46^,^47^,^48^. For our study, we prepared films containing mixtures of synthetic eumelanin and pheomelanin and probed these films using three different potential inputs as illustrated by the upper panels in Fig. 9 (see Figure S5 of the Supplementary Information for further details). As indicated by the middle panels in Fig. 9, outputs were analyzed by integrating the currents to determine the total charge transferred during reduction (*Q*_*Red*_) and oxidation (*Q*_*Ox*_), and then creating a reduction to oxidation ratio (Redox Ratio). The expectation is that a sample with greater redox-based pro-oxidant activity would have a higher Redox Ratio as it would draw more electrons from the electrode.

In the first test, we performed cyclic voltammetry (e.g., as in Fig. 8) and analyzed the results as illustrated by the upper panel in Fig. 9a. For the synthetic melanin mixtures, the results in bottom panel of Fig. 9a show a linear increase in Redox Ratio with the fraction of synthetic pheomelanin. This observed trend is expected from the greater pro-oxidant activity of synthetic pheomelanin. Superimposed on the linear plot in Fig. 9a are horizontal lines for the results from the natural melanin samples which show that the Redox Ratio for pheomelanin is greater than that for eumelanin. Interestingly, the intersection of the horizontal line for pheomelanin with the results from the synthetic mixtures suggests that the content of eumelanin in the natural pheomelanin sample is approximately 40% which is in agreement with chemical analysis of red human hair^39^.

In the second test, the upper panel in Fig. 9b shows that we imposed stepwise potential inputs to each of the melanin film coated electrodes for 5 min and measured the charge transfer for both the step change to the reducing conditions and to the oxidizing conditions (analogous to experiments in Fig. 7). The plot in Fig. 9b again shows that for the synthetic melanin mixtures, the Redox Ratio increased linearly with the fraction of synthetic pheomelanin. The results for the natural melanins (horizontal lines) again show the Redox Ratio for pheomelanin is larger than for eumelanin.

The final test was performed by cycling the potential 10-times between −0.1 V and 0.8 V to probe the more oxidative redox potential range (analogous to experiments in Fig. 5). As indicated by the middle panel in Fig. 9c, we averaged the charge transferred from last three cycles and calculated the Redox Ratio. Consistent with the previous two tests, the Redox Ratio increased linearly with the fraction of synthetic pheomelanin in the film, and the natural pheomelanin had a higher Redox Ratio than natural eumelanin.

In conclusion, results for the natural melanins are qualitatively consistent with those for the synthetic models: the melanins are redox-active and pheomelanin has a greater redox-based pro-oxidant activity.

## Discussion

The first conclusion from this reverse engineering study is that the melanins all displayed redox-cycling capabilities: soluble electron shuttles can rapidly (sec-min) and repeatedly exchange electrons with the melanins in both oxidative and reductive directions. Typically, redox-activities of melanins are studied in one direction. Several studies have reported that melanins (or melanin-like models) are redox-active^23^,^49^ and can be reduced by accepting electrons from biological reductants (e.g., NAD(P)H and glutathione)^9^,^10^. Alternatively, melanins have been shown to be oxidized and donate their electrons to biologically-relevant oxidants such as O_2_^2^,^50^,^51^,^52^,^53^ to generate reactive oxygen species (ROS). Here we used electrochemical methods as a simple means to repetitively probe the redox-activities in both oxidative and reductive directions. Importantly, the ability of melanins to redox-cycle both oxidatively and reductively indicates a “catalytic” activity that promotes the transfer electrons from reductants to oxidants. Such catalytic-activities have been suggested previously^9^,^10^,^49^,^54^. In fact, earlier work reported that melanin’s electron-transfer abilities are so great that it is essentially a conducting polymer^19^,^55^,^56^,^57^. While these observations are technologically exciting, their biological relevance is uncertain.

The important biological implication of melanin’s redox-cycling catalytic activity is that it could substantially influence biological redox homeostasis. Cells use several biological redox couples (e.g., NAD(P)H/NAD(P), GSH/GSSG, and CysS/CysSS) which are generally believed to be kinetically “insulated” (e.g., these redox couples are not in equilibrium within their microenvironment and not in equilibrium with each other)^58^,^59^,^60^. If melanins act as non-specific catalysts for electron transfer, then this activity would disrupt the kinetic insulation and promote equilibration among these various biological redox species. Such catalytic activity would tend to drive redox conditions to values very near melanin’s redox potential and this redox-buffering could substantially disrupt cellular redox balances.

The second important conclusion from this study is that pheomelanin has greater redox-based pro-oxidant activity than eumelanin. We used synthetic models and several approaches to reveal that pheomelanin has a more oxidative redox potential while studies with the more complex natural pheomelanins demonstrated signatures characteristic of a greater redox-based pro-oxidant activity. Our observed oxidative potential is consistent with the redox-potential of 1,4-benzothiazines arising from oxidative cyclization of 5-S-cysteinyldopa^43^,^61^. Also, the redox-ranges observed here are quantitatively similar to values reported from photoelectron emission microscopy: −0.2 V vs NHE (≈−0.4 V vs Ag/AgCl) for eumelanin and +0.5 V vs NHE (≈+0.3 V vs Ag/AgCl) for pheomelanin^15^,^18^. The important implication of pheomelanin’s more oxidative redox potential (compared to eumelanin) is that it would tend to buffer redox conditions to a more positive (more oxidative) potential. The thermodynamic plot comparing eumelanin and pheomelanin is shown in Scheme S1 of the Supplementary Information. This scheme allows us to reconcile previous observations that show pheomelanin can serve as a redox catalyst accepting H-atoms from a broader variety of physiologically relevant reducing agents with respect to eumelanin and transferring these electrons to oxygen with the associated generation of ROS.

In conclusion the results from the present study provide the first evidence that pheomelanin is thermodynamically allowed to induce consumption of antioxidants critical for maintaining the cellular redox homeostasis thus sustaining a chronic oxidative stress condition that is generally held as the underlying cause of melanoma development in red hair phenotypes.

## Materials and Methods

### Chemicals

The following were purchased from Sigma-Aldrich: chitosan, 1,1′-ferrocenedimethanol (Fc), Ru(NH_3_)_6_Cl_3_ (Ru^3+^), and K_3_IrCl_6_ (Ir^3+^). The water (>18 MΩ) used in this study was obtained from a Super Q water system (Millipore). Chitosan solutions (1%, pH 5.5) were prepared by dissolving chitosan flakes in HCl to achieve a final pH of 5–6. The solutions of mediator were prepared in phosphate buffer (0.1 M; pH 7.0). Previously described methods were used to prepare the synthetic eumelanin^62^ and pheomelanin^35^. The natural melanin samples were purified from human hairs as previously reported^9^.

### Preparation of melanin-chitosan film coated electrode

To prepare films with entrapped melanin samples, we first dispersed the melanin samples (synthetic or natural; 5 mg/mL) in water sonicated for 2 hours, the supernatant fraction was mixed with a chitosan solution (pH 5.5) to make melanin-chitosan suspension (final chitosan concentration 0.5%). Aliquots (20 μL) of these melanin-chitosan suspensions were spread onto the electrode and dried under vacuum. After drying, these film coated electrodes were immersed into a 0.1 M phosphate buffer solution (pH 7.0) to neutralize the chitosan and form an insoluble hydrogel film (chitosan is insoluble after neutralization). The melanin content in synthetic and natural melanin chitosan film was estimated by measuring absorbance at 500 nm^34^ against solutions of the pigments obtained in alkali under controlled atmosphere and measured after dilution at pH 5.5. The synthetic eumelanin and pheomelanin content in chitosan films was estimated as 91 and 104 μg/mL, respectively; the natural eumelanin and pheomelanin content in chitosan films was estimated as 248 and 484 μg/mL. More detailed assay conditions are described in Supplementary Information.

### Instrumentation

Raman spectra were obtained using a Jobin Yvon LabRam HR Raman Spectroscopy. Electrochemical measurements (cyclic voltammetry and chronocoulometry) were performed using a three electrode system with Ag/AgCl as a reference electrode, Pt wire as a counter electrode, and a gold disk electrode (r = 1 mm) as a working electrode (CHI Instruments 6273C and CHI420a electrochemical analyzer). Air was excluded by purging N_2_ during the electrochemical experiment.

## Additional Information

**How to cite this article**: Kim, E. *et al*. Reverse Engineering Applied to Red Human Hair Pheomelanin Reveals Redox-Buffering as a Pro-Oxidant Mechanism. *Sci. Rep*. **5**, 18447; doi: 10.1038/srep18447 (2015).

## Supplementary Material

Supplementary Information

## Figures and Tables

**Table 1:**
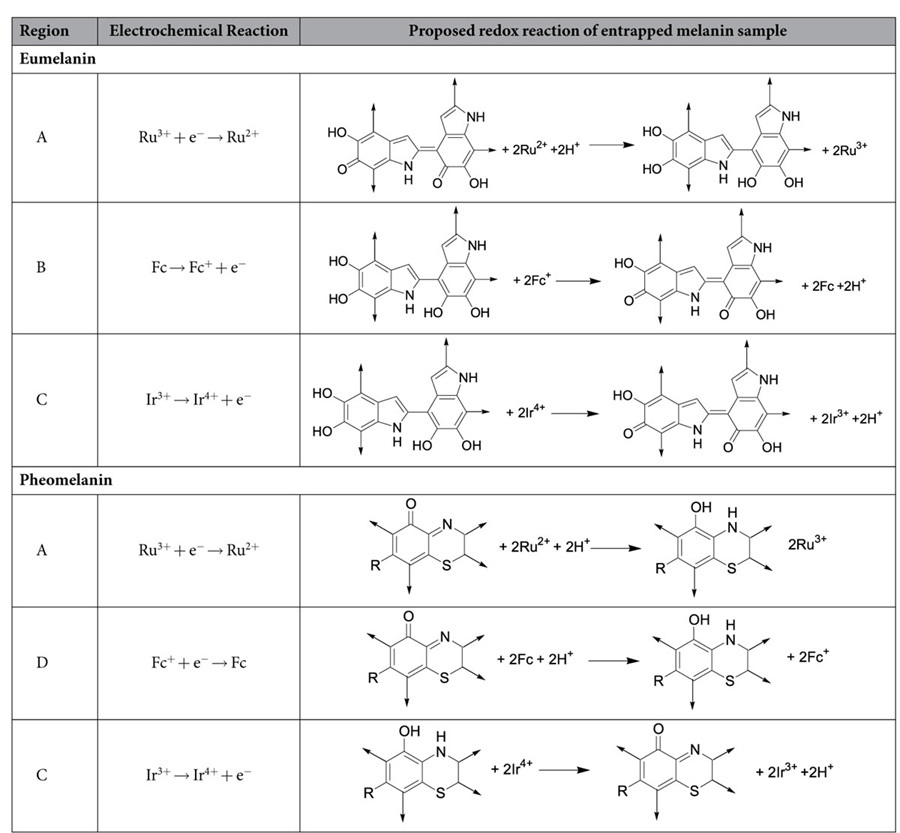
Proposed redox reactions of melanins.
