# Supplementary material for: Reverse Engineering Applied to Red Human Hair Pheomelanin Reveals Redox-Buffering as a Pro-Oxidant Mechanism
Source: Sci Rep. 2015 Dec 16;5:18447. doi: 10.1038/srep18447 (PMC4680885; doi:10.1038/srep18447)
Supplement: Supplementary Information [file srep18447-s1.pdf]

Supplementary Information

REVERSE ENGINEERING APPLIED TO RED HUMAN HAIR PHEOMELANIN REVEALS REDOX-BUFFERING AS A PRO-OXIDANT MECHANISM

Eunkyoung Kim, Lucia Panzella, Raffaella Micillo, William E. Bentley,
Alessandra Napolitano and Gregory F. Payne

Figure S1

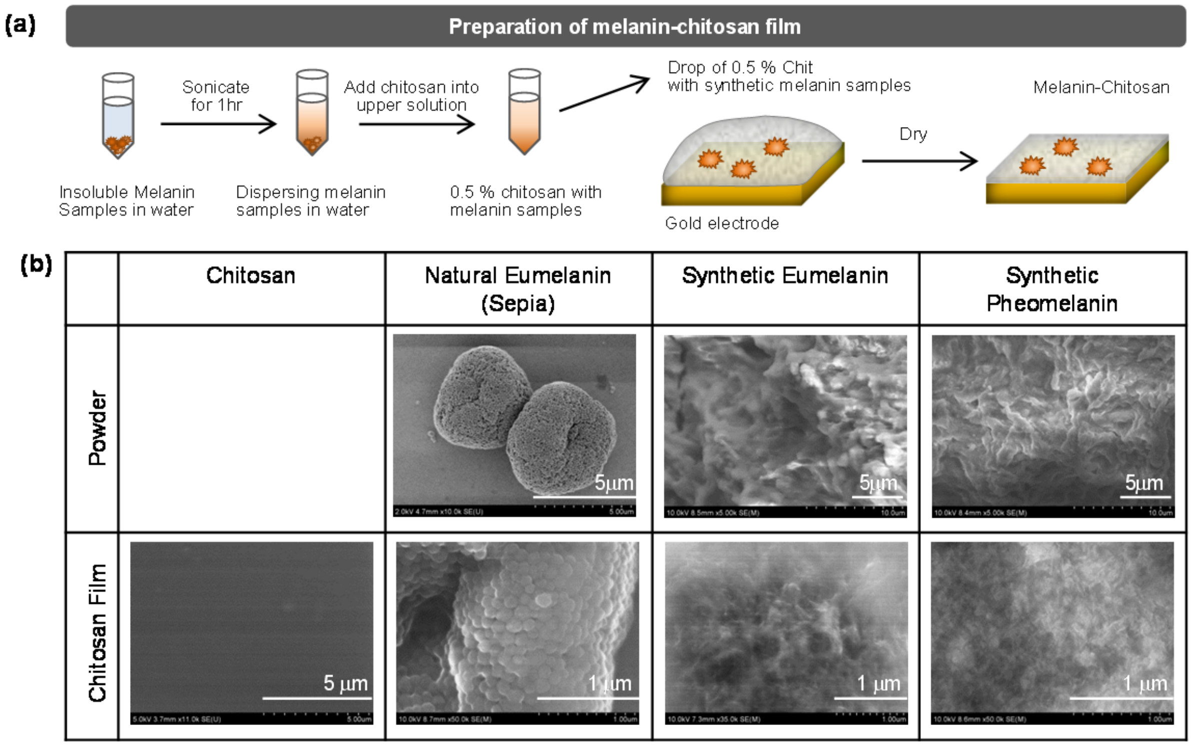

Figure S1. **Melanin-chitosan films.** (a) Procedure to prepare melanin-containing chitosan films at an electrode interface. (b) SEM images of melanin powders (top images) and melanin-containing chitosan films (bottom images).

Our electrochemical method probes melanin samples that are entrapped within a hydrogel film adjacent to an electrode surface. **Figure S1a** illustrates the experimental method to prepare these melanin entrapped films. First, melanin samples (5 mg/ml) were spread in water and then these aqueous melanin-spread suspensions were sonicated for 2 hours to decrease the melanin particle size. After allowing particulates to settle, we mixed the supernatant from the sonicated melanin-water dispersions with a chitosan solution (0.9 %, pH 5.5) to make melanin-chitosan suspension (final chitosan concentration 0.5 %). Aliquots (20 µL) of these melanin-chitosan suspensions were spread onto the electrode and dried under vacuum. After drying, these melanin-chitosan film coated electrodes were immersed into a 0.1 M phosphate buffer solution (pH 7.0) to neutralize the chitosan and form an insoluble hydrogel film (chitosan is insoluble after neutralization).

The melanin samples and their melanin-chitosan films were observed with scanning electron microscopy (SEM). To compare synthetic eumelanin with a natural eumelanin, we prepared a eumelanin chitosan film using melanin from *Sepia officinalis* (cuttlefish), which serves as the common model of natural eumelanin.[1] In the top images of **Figure S1b**, Sepia eumelanin shows a spherical-shaped particle (2μm-10μm), which is composed of aggregated granules (≈100-200 nm) as reported previously.[2] The SEM images of synthetic eumelanin and synthetic pheomelanin do not display the hierarchical structure of Sepia melanin, but rather show a randomly aggregated structure. The SEM image at the bottom left of Figure S1b show that the unmodified chitosan film is flat and featureless. In contrast, the melanin-chitosan films show a rough morphology consistent with the particulate morphology of the melanin samples. Further, the SEM images at the bottom of Figure S1b are less clear than those at the top suggesting that chitosan effectively coats the samples.

Quantification of melanin in the melanin chitosan film

For evaluation of the melanin content, the pigments were dissolved in 0.1 M NaOH that had been purged with argon for at least 15 min at 1 mg/mL. After completely dissolving, melanin solutions were prepared by dilution into 0.2 M phosphate buffer at pH 5.5 and the spectra were registered in the 300 to 700 nm range. A calibration curve was built based on the absorption at 500 nm. The spectra of the melanin containing chitosan films were as well registered and the content of melanin was evaluated based on the absorption at 500 nm against the calibration curve obtained for the reference pigment.

Figure S2

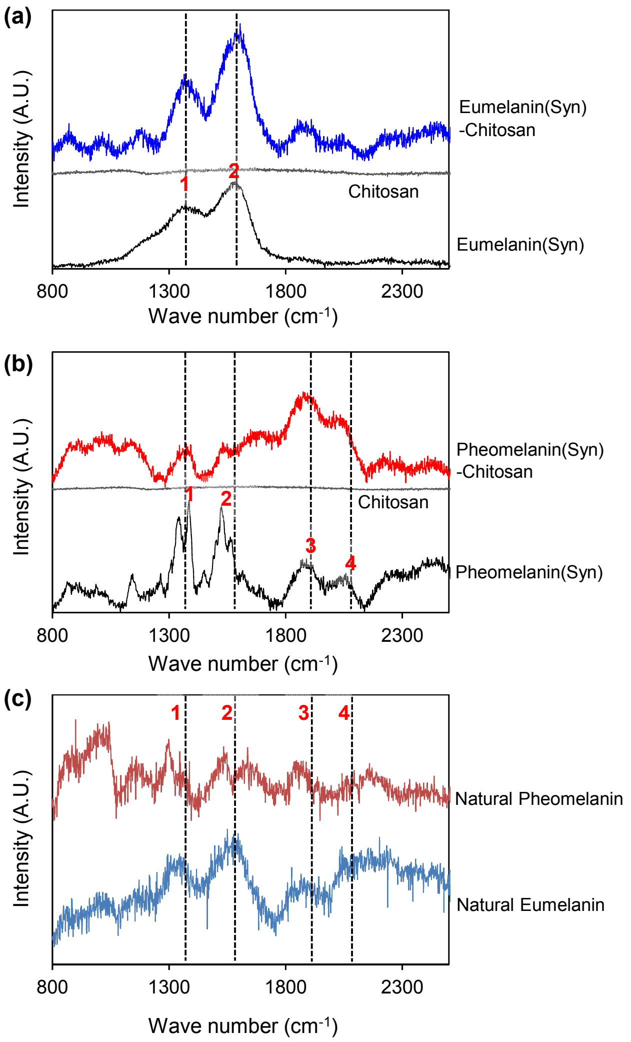

Figure S2. **Raman characterization of melanin samples and melanin-chitosan films.** (a) Synthetic eumelanin. (b) Synthetic pheomelanin. (c) Natural melanins.

Chemical characterization of the melanin-chitosan films were performed using Raman spectroscopy. **Figure S2a** shows that the spectrum of synthetic eumelanin powder has two closely-spaced characteristic bands.[4] Band 1 (1360 cm^{-1}) and band 2 (1580 cm^{-1}) that have been attributed to the linear stretching of the C-C bonds within the aromatic rings and the in-plane stretching of the aromatic rings, respectively.[4, 5] These two characteristic bands were also observed when synthetic eumelanin was entrapped in the chitosan film (chitosan shows no bands in this spectral region).

Raman characterization of the synthetic pheomelanin powder and the film-entrapped pheomelanin are shown in **Figure S2b**. Compared to the spectrum for synthetic eumelanin, the Raman spectrum for these pheomelanin samples show two additional prominent peaks above 2000 cm^{-1} (band 3 and band 4). Previous studies suggest these bands are related to overtone or combination bands.[6] These Raman bands were also observed for both the powder and film-entrapped synthetic pheomelanin samples.

Raman spectra of natural melanin samples in **Figure S2c** exhibit features that are consistent with those observed for synthetic pigments though more complex due to the contribution of the protein matrix.[4, 6] In the case of natural pheomelanin bands attributable to eumelanin can be identified,[4] suggesting the presence of a significant eumelanin component in natural pheomelanin.[7]

Figure S3

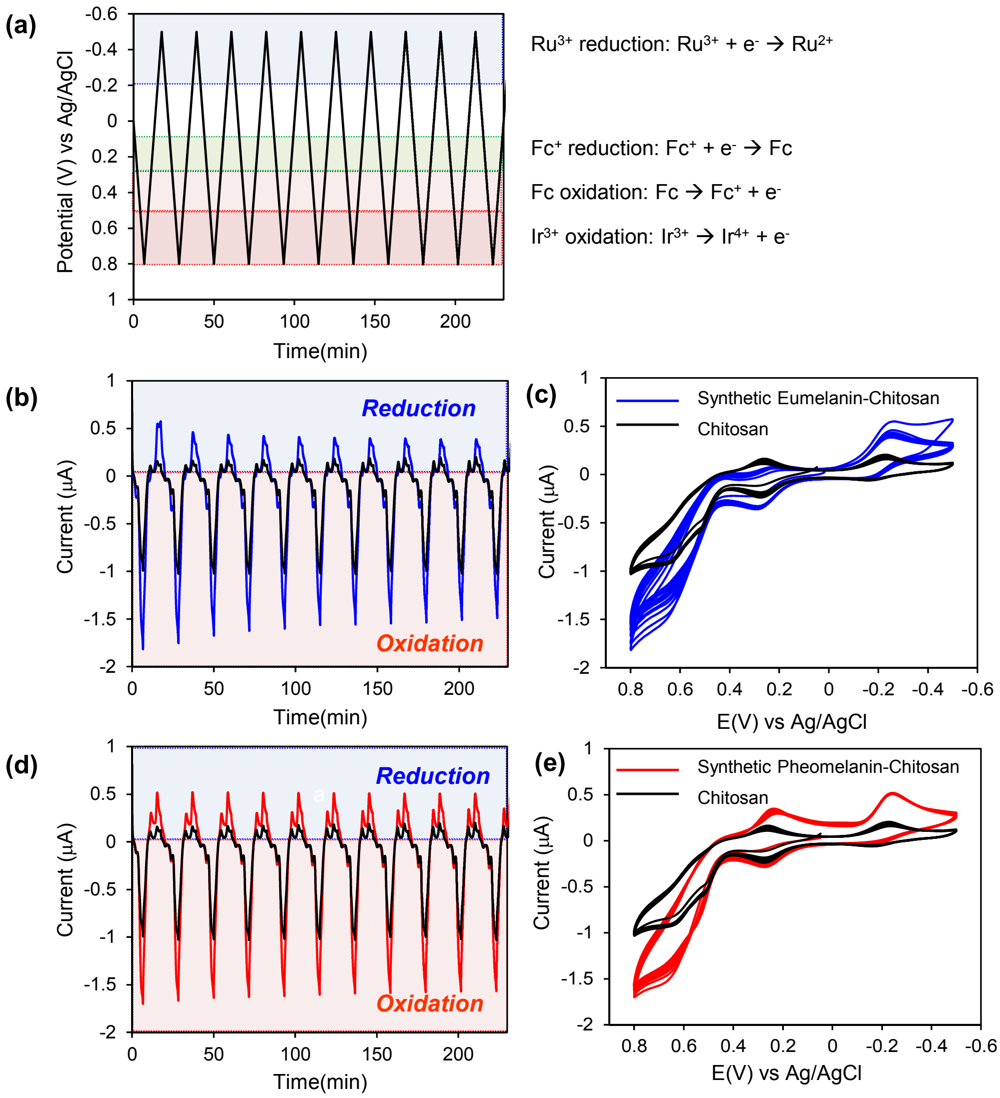

Figure S3. The redox activity of melanin is reversible and steady. (a) Sequence of input voltages used to probe the synthetic melanin potential window. (b) Current output curve and (c) cyclic voltammogram for eumelanin-chitosan film. (d) Current output curve and (e) cyclic voltammgoram for pheomelanin-chitosan film.

As observed in Figure 3 of the manuscript, a multiple-cycled cyclic voltammetry of the synthetic melanin was performed by immersing the film-coated electrodes in a mixed solution of three redox mediators (100 μM Ir^{3+}, 50 μM Fc, and 50 μM Ru^{3+}) and applying a cyclic potential to the underlying electrode (between -0.5 V and +0.8 V at a scan rate 2 mV/s). Figure S3a showed sequence of input voltages of 10 cycled potentials. Figure S3b and Figure S3c showed the

current output and cyclic voltammogram of synthetic eumelanin-chitosan film coated electrode. Compared with chitosan film, the eumelanin-chitosan showed the higher redox current due to the redox-cycling reaction as suggested in Figure 3. Even though the redox current is slightly decreased in the initial cycles, the redox current was kept steady after a few cycles. Figure S3d and Figure S3e showed the current output of pheomelanin-chitosan film, which indicates that the redox current was reversibly switched and kept steady during 10 cycles. Figure S3 supports that the redox state of melanin can be reversibly switched and the conversion is very stable over prolonged times.

Figure S4

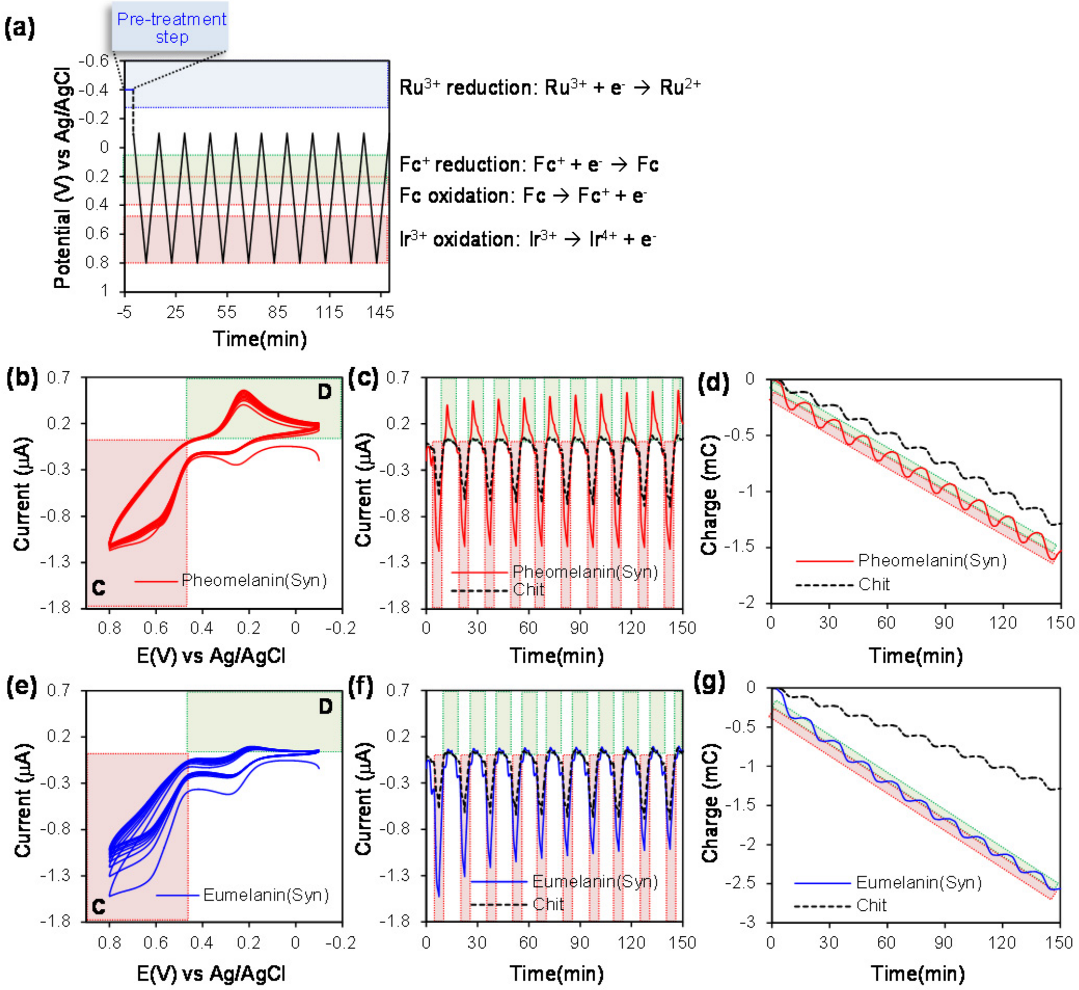

Figure S4. Repeated probing of the pheomelanin potential window for evidence of reversible redox-ctivity. (a) Sequence of input voltages used to probe the pheomelanin potential window. Current output for pheomelanin-chitosan film expressed as (b) cyclic voltammogram, (c) output curve (Current) or (d) output curve (Charge). Current output for eumelanin-chitosan film expressed as (e) cyclic voltammogram, (f) output curve of current or (g) output curve of charge. Pheomelanin's paired amplification of oxidation-reduction currents and the "steady" output indicates pheomelanin redox activity is reversible.

Figure S5

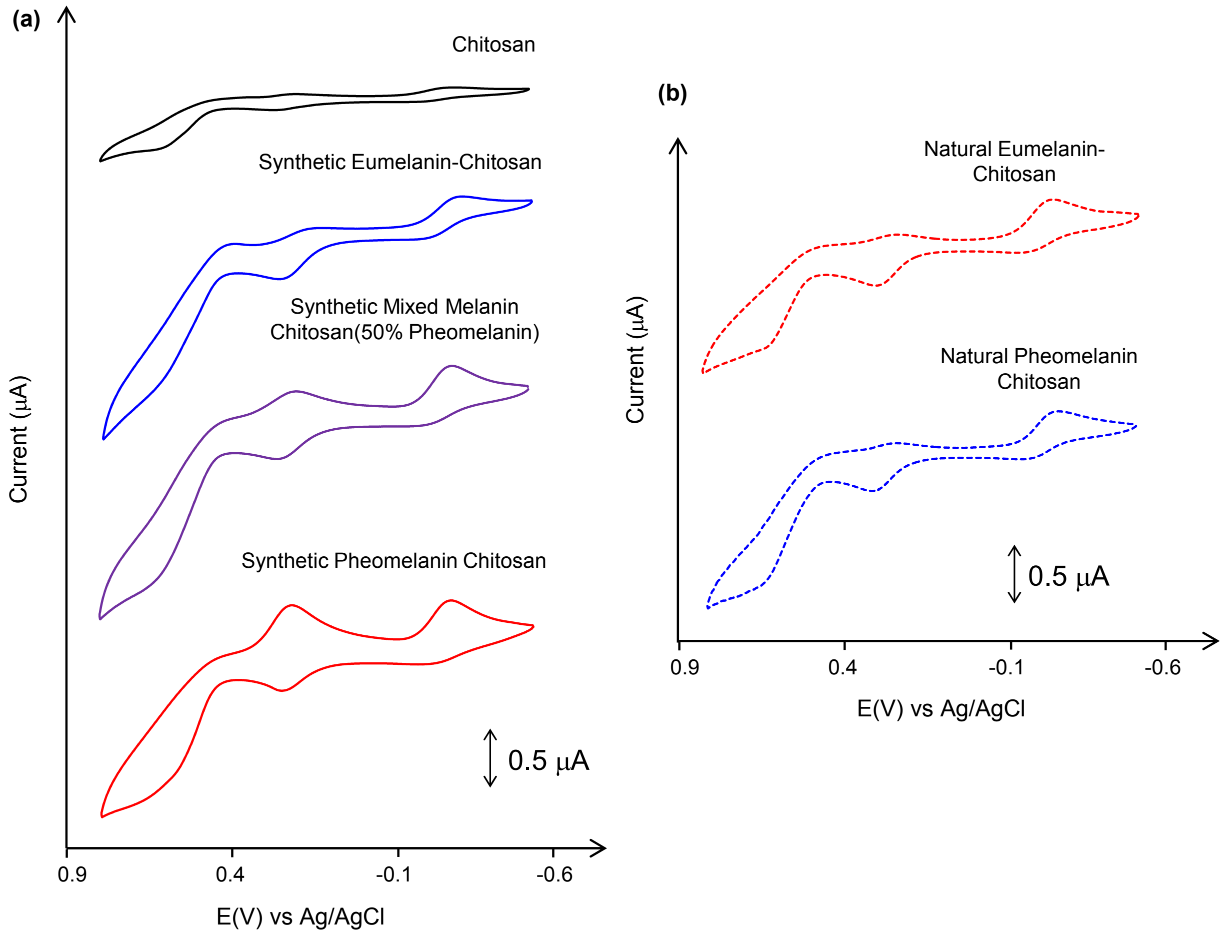

Figure S5. **Different redox activity of melanin dependent on the pheomelanin content.** (a) Cyclic voltammograms of various mixed synthetic melanin-chitosan coated electrodes. (b) Cyclic voltammograms of natural melanin-chitosan coated electrode. All of CVs were measured in a mixed solution of 100 μM $Ir^{3+,}$ 50 μM Fc and 50 μM Ru^{3+} at a scan rate of 2 mV/s.

Scheme S1

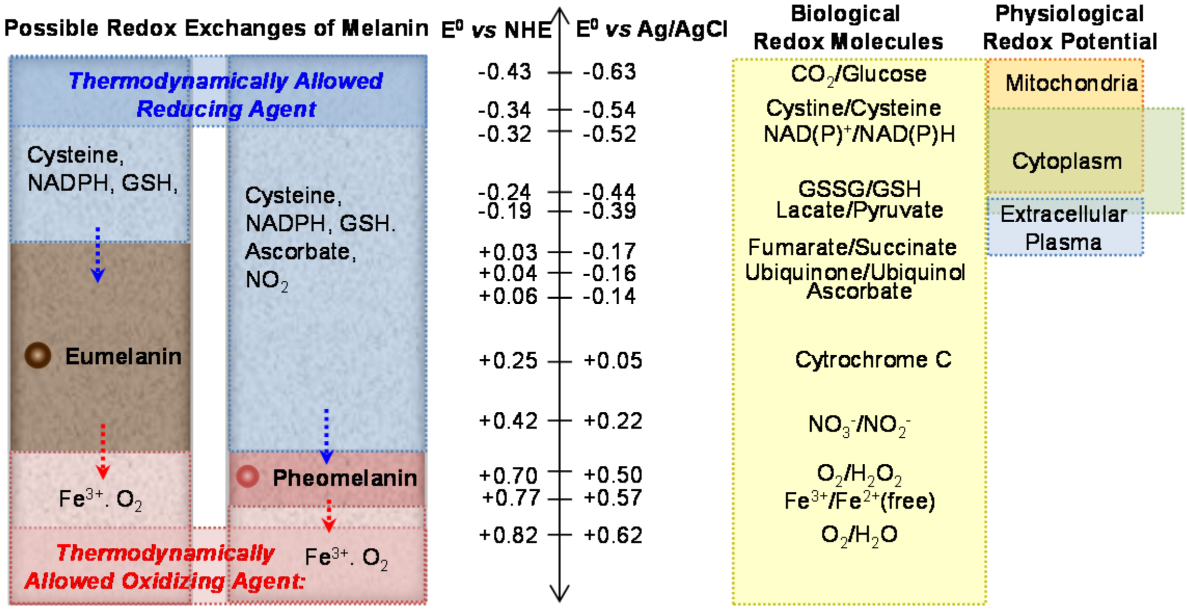

Possible Redox Exchanges of Melanin
E0 vs NHE
E0 vs Ag/AgCl
Biological Redox Molecules
Physiological Redox Potential
Thermodynamically Allowed Reducing Agent
Cysteine, NADPH, GSH,
Eumelanin
Fe3+. O2
Cysteine, NADPH, GSH. Ascorbate, NO2
Pheomelanin
Fe3+. O2
Thermodynamically Allowed Oxidizing Agent:
-0.43 -0.63
-0.34 -0.54
-0.32 -0.52
-0.24 -0.44
-0.19 -0.39
+0.03 -0.17
+0.04 -0.16
+0.06 -0.14
+0.25 +0.05
+0.42 +0.22
+0.70 +0.50
+0.77 +0.57
+0.82 +0.62
CO2/Glucose
Cystine/Cysteine
NAD(P)+/NAD(P)H
GSSG/GSH
Lacate/Pyruvate
Fumarate/Succinate
Ubiquinone/Ubiquinol
Ascorbate
Cytrochrome C
NO3-/NO2-
O2/H2O2
Fe3+/Fe2+(free)
O2/H2O
Mitochondria
Cytoplasm
Extracellular Plasma

References

1. Glass, K. *et al.* Direct chemical evidence for eumelanin pigment from the Jurassic period. *Proc. Natl. Acad. Sci. U. S. A.* **109**, 10218-10223 (2012).
2. Liu, Y. & Simon, J.D. Isolation and Biophysical Studies of Natural Eumelanins: Applications of Imaging Technologies and Ultrafast Spectroscopy. *Pigment Cell Res.* **16**, 606-618 (2003).
3. Wakamatsu, K. & Ito, S. Advanced Chemical Methods in Melanin Determination. *Pigment Cell Research* **15**, 174-183 (2002).
4. Galván, I. *et al.* Raman spectroscopy as a non-invasive technique for the quantification of melanins in feathers and hairs. *Pigment Cell & Melanoma Research* **26**, 917-923 (2013).
5. Huang, Z. *et al.* Raman spectroscopy of in vivo cutaneous melanin. *J Biomed Opt* **9**, 1198-1205 (2004).
6. Galvan, I., Jorge, A., Solano, F. & Wakamatsu, K. Vibrational characterization of pheomelanin and trichochrome F by Raman spectroscopy. *Spectrochim. Acta. A Mol. Biomol. Spectrosc.* **110**, 55-59 (2013).
7. Ito, S. & Wakamatsu, K. Diversity of human hair pigmentation as studied by chemical analysis of eumelanin and pheomelanin. *J. Eur. Acad. Dermatol. Venereol.* **25**, 1369-1380 (2011).
